# Design and simulation of a low-energy atomic silicon quantum-dot circuit with potential in internet of things applications

**DOI:** 10.1038/s41598-025-12009-3

**Published:** 2025-09-30

**Authors:** Hadi Rasmi, Mohammad Mosleh, Nima Jafari Navimipour, Seyed-Sajad Ahmadpour, Mohammad Kheyrandish

**Affiliations:** 1 Department of Computer Engineering, Dez.C., Islamic Azad University, Dezful, Iran; 2 Department of Computer Engineering, Ta.C., Islamic Azad University, Tabriz, Iran; 3 Future Technology Research Center, National Yunlin University of Science and Technology, Douliou, Yunlin, Taiwan; 4 Research Center of High Technologies and Innovative Engineering, Western Caspian University, Baku, Azerbaijan; 5 Department of Computer Engineering, Faculty of Engineering and Natural Sciences, Istanbul Atlas University, 34408 Istanbul, Türkiye

**Keywords:** Nanotechnology, Logical gates, Comparator circuit, Atomic silicon dangling bond (ASDB), Energy science and technology, Engineering, Materials science, Nanoscience and technology

## Abstract

This paper addresses critical issues such as leakage and heating in Internet of Things (IoT) circuits by exploring alternatives beyond CMOS technology. Atomic silicon dangling bond (ASDB) technology emerges as a promising substitute for executing nanoscale logic circuits, particularly for IoT applications requiring compactness, efficiency, and energy optimization. We propose a Hammer-shaped design for ASDB basic gates to enhance circuit stability and optimality, which is vital for the reliable operation of IoT systems. we demonstrate a new ASDB one-bit comparator circuit to highlight the practical application of the proposed design, which is crucial for real-time data processing in smart homes, industrial automation, health monitoring, connected vehicles, environmental sensors, and smart grids. By integrating high-performance comparator circuits, IoT networks gain improved accuracy and reduced latency, enabling advancements in energy management and wearable electronics. Simulation results highlight significant improvements, including a 33% enhancement in occurrence, 27.% in energy efficiency, 56% resistance to DB omission, and 51% in extra DB deposition.

## Introduction

The use of CMOS technology in the design of complex circuits has become prevalent in Internet of Things (IoT) applications, especially with significant advances in lithography that enable the implementation of computational circuits at the nanoscale^1,2^. However, at dimensions below 4 nm, this technology faces critical challenges, including heat generation, high power consumption, and leakage^3–6^. Among these, VLSI designers have researched a few alternative technologies: Fin-shaped field-effect transistors (FinFET)^7^, Carbon nanotube field-effect transistors (CNTFET), Tuned field-effect transistors (TFET), Single electron transistors (SET), Quantum-dot cellular automata (QCA), and atomic silicon dangling bond (ASDB) technology. These may provide potential solutions for the challenges faced by CMOS technology in IoT devices^8^.

As previous studies have shown, nanotechnology could be an attractive alternative to CMOS because of its more advantageous properties, including higher switching speed, smaller size, lower power consumption, and better thermal stability. Some researchers shifted their interest in ASDB nanotechnology, which is promising to create circuit design below 4 nm^9^. Through ASDB technology, the circuit could be designed in the form of accurately placed dangling bond (DB) atoms. These small DBs allow for the complex circuit creation on an H-Si (100) surface, having intricate and specific patterns that are so important in furthering the use of the Internet of Things applications in things like smart home devices, wearables, environmental sensors, smart grids, and connected vehicles^10^.

**Fig. 1:**
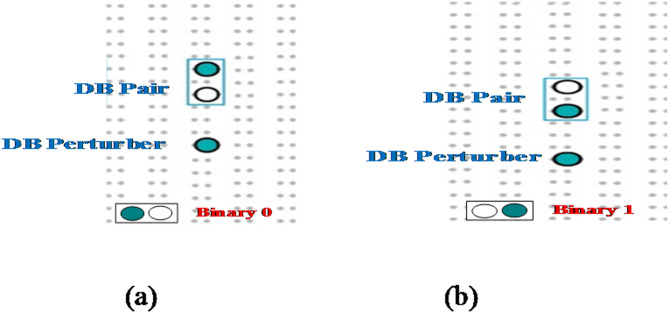
Frequency shift (∆*f*) images (a) DB pair with a right perturber, defined as binary 0, (b) DB pair with a left perturber, defined as binary 1^15^.

ASDB is formed when a silicon atom lacks the four regular bonding partners, this results in what is know as a dangling bond (DB)^11^. When these DBs are placed on the surface, they disrupt the periodicity of the crystal and lead to the rearrangement of the surface atoms. The silicon dangling bond on the H-Si (100) surface forms a pattern. Such patterns can be used to build circuits. Their special electronic properties make them good for future devices. By providing new patterns, new circuits can be designed, potentially revolutionizing digital circuit design and revolutionizing metal-oxide-semiconductor (CMOS) technology^12^. Atomic silicon dangling bonds (ASDBs) work well with low-power strategies based on quantum dots. The ASDB approach uses binary logic, without current-based technologies. These ASDBs fit into the existing architecture, opening up possibilities for the future of Nanoelectronics. Atomic silicon dangling bonds (ASDBs) have created a new way of thinking about nano computing. ASDB logic uses coulombic interactions instead of electrical energy^13 ^. This novel approach reduces energy consumption and can process things quickly, even at terahertz frequencies. The ASDB technology is being used more and more^14^. Researchers can now create individual dangling bonds on a hydrogen-passivated silicon surface at the single-atom scale, using scanning tunneling microscopy (STM). These ASDBs act as quantum dots, representing logical states and performing Boolean operations within the physical scaling limit^14^. A single dangling bond (DB) in ASDB technology emerges when a hydrogen atom (H) is replaced, turning it into a quantum dot. In the past 20 years, researchers have made big advances in nanotechnology, especially in controlling individual DB atoms for changing quantum states. This involves using STM to create quantum holes and arrange DB atoms. Single DBs are formed by replacing a hydrogen atom. On the silicon surface, these DBs have three different charges: negative, neutral, and positive. Each pair of DBs can hold a negative charge. This principle is used to define binary “0” and “1.” An electrostatic perturber is introduced to the left or right side of a DB pair (see Fig. 1)^ 10^.

The energy-efficient, high-performance circuits required by next-generation IoT systems can be developed using the ASDB technology, thus addressing the limitations being faced by conventional CMOS technology in the nanoscale regime^14^. This shift towards the ASDB technology will further improve performance in IoT devices and open new application areas for smart cities, industrial automation, health monitoring, and remote sensing^16^. Further, the integration of ASDB technology into the IoT ecosystem can be expected to enhance data processing, energy management, and overall system reliability, therefore becoming a fundamental building block for future developments^ 11^.

On the other hand, comparator circuits are fundamental components of electronic systems and find broad applications in IoT. A comparator serves as an electronic circuit that compares two voltage levels for an output based on which of the two inputs is higher^17^. These circuits have wide applications, ranging from signal conditioning, where they convert analog signals to digital ones, to threshold detection, which forms an important part of things like alarm systems and temperature monitoring, among others^9^. In IoT systems, comparators facilitate the process of data acquisition by translating information retrieved from sensors, most of the time in analog form, into digital signals that a microcontroller or other device is capable of processing^10^.

Comparators also play a significant role in IoT while enabling real-time monitoring and responding instantly to changes in the environment, which are crucial in applications ranging from smart homes to industrial automation^1^. They will also contribute to energy efficiency. Comparators can let IoT devices go into low-power mode, turning them on only if certain conditions set by the system are met and hence conserve energy. This can again be related to IoT because of their functionality as a means of sensor data integration and automated process controllers that provide continuous feedback for the purposes of sustaining processes within desirable levels^14^.

ASDB nanotechnology has several important aspects: how to position DB atoms on the silicon surface, withstanding potential synthesis defects^10^, and stability of output against temperature variations^10^. Selecting an appropriate design pattern can effectively address many of these challenges.

So far, ASDB researchers have introduced four different patterns, named Y-shape^16^, T-shape^13^, P-shape^10^, and Bar-shape^12^, for circuit design. These patterns serve as the basis for designing ASDB logic gates. Their stability and adaptability make them ideal candidates for creating reliable and efficient circuits.

. To date, only a few studies have investigated this technology, as reviewed below.

In a groundbreaking achievement, Eigler and Schweitzer pioneered the precise control and manipulation of individual atoms on a silicon surface^18^. Their work marked the first time that scientists could intentionally move atoms on silicon surfaces, opening exciting possibilities for nanoscale computing.

In 2018, Huff et al. proposed an OR logic gate based on DBs on a hydrogen-inactivated silicon surface^15^. These DBs, which represent unpaired electrons on the silicon surface, were shown to be programmable during a process. Their behavior can be harnessed to design and implement basic logic circuits. That research represented a significant step toward harnessing atomic-scale phenomena for practical applications in computing and technology. In the same year, as the pioneering work by Achal et al., researcher made significant strides in designing robust and editable memories based on DBs^17^; those memories could read and write up to 192 bits of data. To accomplish this, they precisely engineered a network of dangling bonds (DBs) on the surface of hydrogen-inactivated silicon (H-Si 100).

In 2020, Ng et al. introduced a Y-shaped pattern for designing basic logic circuits^19^. Their approach involved utilizing a computer-aided design (CAD) tool called Silicon Quantum Atomic Designer (SiQAD). That tool allowed them to create and observe DB circuits prior to fabrication. Also, in same year Bahar et al. introduced a T-shape pattern for circuit design within the realm of ASDB Nanotechnology. Notably, they presented two distinct gates: a three-input majority voter (MV3) gate and a three-input XOR (XOR3) gate^13^.

In 2023, Rasmi et al. proposed a new structure involving triangles and rhombuses for designing MV3 and XOR3 gates^11^. Furthermore, they successfully implemented a full adder using their innovative structures. In 2023, Ahmad pour et al. contributed a tolerant MV3 gate, specifically designed for constructing a tolerant full-adder^9^. Their gate design demonstrated robustness against potential defects, including DB omission, extra DB deposition, and DB misalignment. Also, in 2023, Drewniok et al. introduced a method for designing ASDB gates with the fewest possible SiDBs required for a specific Boolean function. Their streamlined approach accelerated the progress in nanotechnology by minimizing gate cost and simplifying ASDB circuit designs.

In 2024, for the first time, Rasmi et al. implemented the robust subtractor circuit using the introduced P-shaped pattern gates^10^, Their proposed circuits had suitable conditions against possible defects, such as DB omission, extra DB deposition, and DB misalignment. Also, in the same year, Rasmi et al. presented a 2:1 multiplexer using a Bar-shaped pattern^12^.

This paper first proposes a novel and efficient design pattern called the Hammer-shape arrangement. Next, some basic gates, including AND, NAND, OR, NOR, XOR, and XNOR, are designed based on the pattern introduced in ASDB. Finally, using the suggested ASDB gates, an ASDB one-bit comparator circuit is introduced. Comparator circuits play a pivotal role in designing microprocessors^16^. They are essential for comparing digital values and determining their relationships, whether one value is more significant than, less than, or equal to another.

This research work will enhance the performance of comparator circuits with a view to enhancing the overall IoT system for better performance in data processing with a minimal quantity of energy use and extension in operational lifespan in applications relating to smart homes, health care, industrial IoT, and environmental monitoring^15^.

The rest of the paper is organized as follows: In section “Proposing ASDB basic gates”, a novel and efficient arrangement pattern of DB atoms, called the Hammer-shaped pattern, will be discussed, and then basic logic gates and comparator circuits in ASDB will be presented. A new efficient ASDB comparator circuit using the proposed ASDB gates has been introduced in section “Proposed ASDB comparator for IoT systems”. The simulation results are given in section “Simulation results”. Finally, the paper ends with a conclusion and future works in section “Conclusion”.

## Basic gates in ASDB

**Fig. 2:**
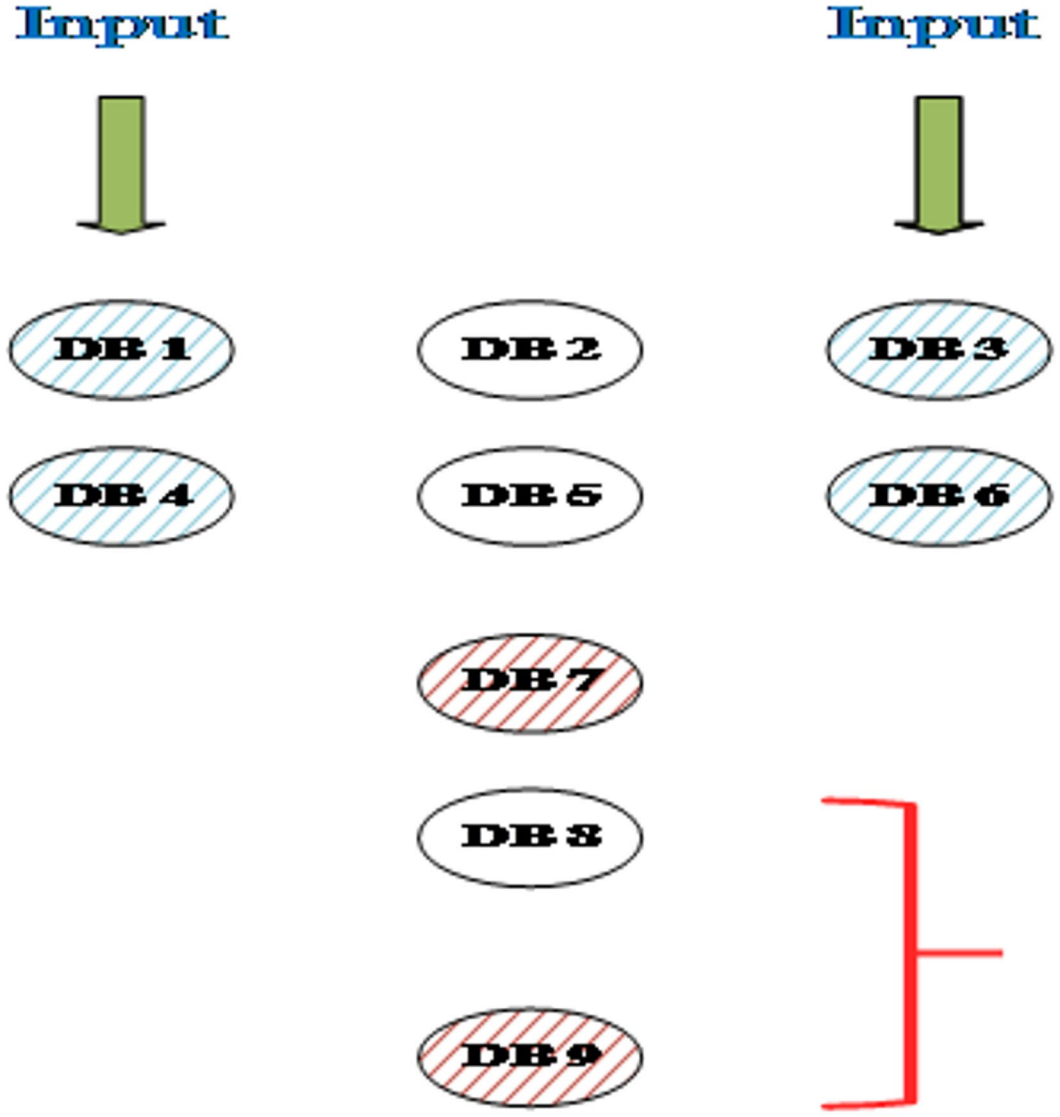
Arrangement of DB atoms in the suggested Hammer-shape pattern.

In this section, the design of basic gates using the proposed Hammer-shaped pattern to arrange DB atoms on the silicon surface is discussed. The arrangement of DB atoms in the introduced hammer shape is illustrated in Fig. 2.

**Fig. 3:**
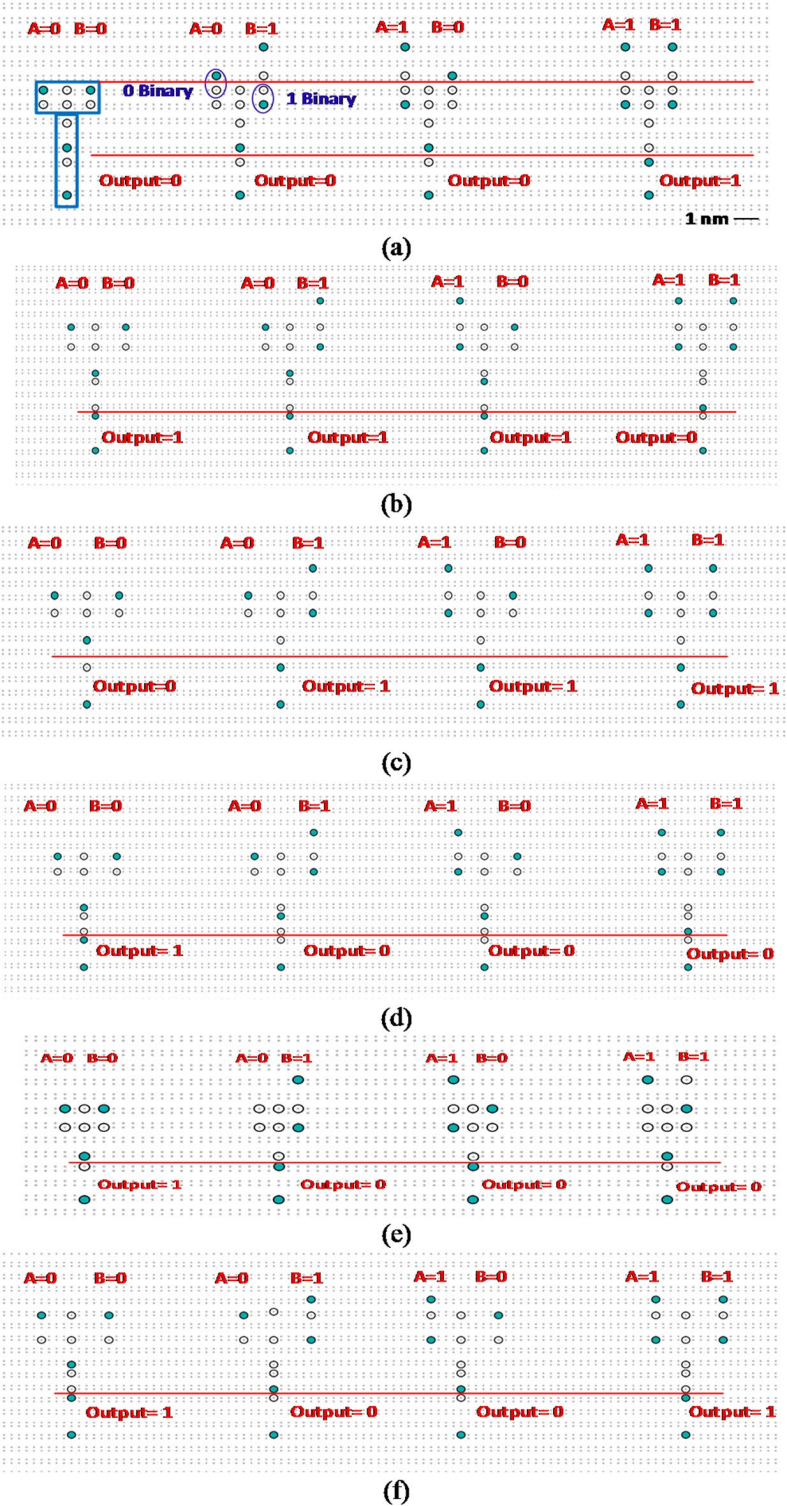
The proposed ASDB layouts for (a) AND, (b) NAND, (c) OR, (d) NOR, (e) XOR, and (f) XNOR gates using SiQAD with ${\epsilon _r}$ = 5.6 and ${{{\varvec{\uplambda}}}_{TF}}$= 5 nm.

The ASDB layouts for six primitive gates, AND, NAND, OR, NOR, XOR, and XNOR, designed using the proposed Hammer-shape pattern with four different input vectors, are illustrated in Fig. 3. In order to test and confirm the proposed ASDB gates, the powerful tools SiQAD has been used. The required configurations for the simulation engine are considered as λ_TF_ > 5 nm and ε_r_ > 5.6; where λ_TF_ and ε_r_ represent Thomas-Fermi screening length and dielectric constant of the silicon surface, respectively^19^.

## Proposed ASDB comparator for IoT systems

A comparator is one of the essential components in a digital circuit, especially in IoT and wireless systems^20^. It compares two voltage levels and provides an output signal according to which of the inputs has a higher level. It is a fundamental process in most decision-making activities in data processing^20^. In the structure of the printed device of the IoT architecture, comparators are basic blocks within the MCU^21^. They process the signals from the temperature and humidity detecting modules for real-time monitoring and decision-making, then send them wirelessly using the BLE module. Efficiency is one of the major concerns for any IoT system to be sustainable^8^. Thus, comparator circuit design with low energy consumption, compact footprint, and minimum complexity is highly essential. It can improve overall productivity and the lifetime of the printed IoT system, with reduced heat generation, by optimizing these factors. The design will include some elements that comparators cannot avoid, such as voltage references, hysteresis controls, and output stages^8,20,21^. All these components will interact in such a way as to create the desired functionality, thus making precise and trustworthy comparisons of sensor data possible. Additionally, comparators make an IoT device stronger for quick responses to changes in the environment and are suitable for applications like smart home automation^8^. Minimizing energy consumption, reducing the occupied area, and enhancing the responsiveness of an IoT device- The proposed printed system aims to optimize the development of efficient comparator circuits^20^.

**Fig. 4:**
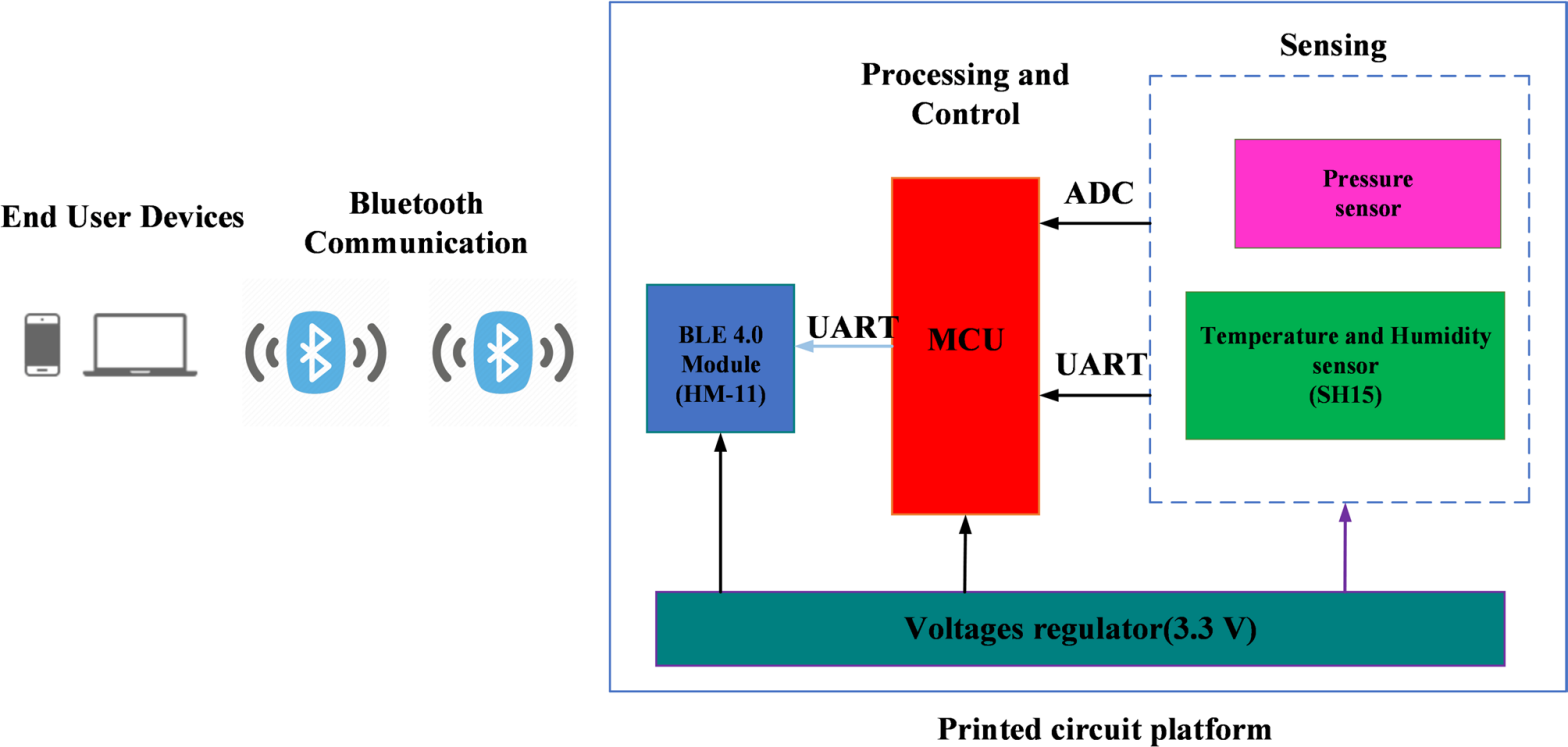
Printed device structure and components^20^.

Thus, this can also help bring sustainability and longevity to the wireless IoT system, allowing it to move towards more reliable operations with less consumption of energy or environmental degradation^20^. Figure 4 shows the structure and composition of the printed device, giving insight into how it will look in the comparator circuit of the system architecture^20^. This correlation shows how comparators can play a significant role in enhancing performance and power efficiency in IoT systems for more practical applications^20^.

A comparator is a crucial combinational logic circuit that compares two numbers and is widely used in the core of microprocessors. This circuit receives two inputs, A and B, and results in three outputs: A less than B (A < B), A equal to B (A = B), and A greater than B (A > B). The truth table of the single-bit comparator circuit is given in Table 1.

**Table 1:** Truth table of single-bit comparator.

Inputs		Outputs		
A	B	f1	f2	f3
0	0	0	1	0
0	1	1	0	0
1	0	0	0	1
1	1	0	1	0

According to Table 1, the output equations resulted as follows:

1 $$A\,<\,B:~f1=\bar {{\varvec{A}}}{\varvec{B}}$$

2 $$A\,=\,B:{\text{ }}f2=\bar {{\varvec{A}}}\bar {{\varvec{B}}}+{\varvec{A}}{\varvec{B}}={\text{ }}A{\text{ }} \odot {\text{ }}B$$

3 $$A\,>\,B:~f3={\varvec{A}}\bar {{\varvec{B}}}$$

**Fig. 5:**
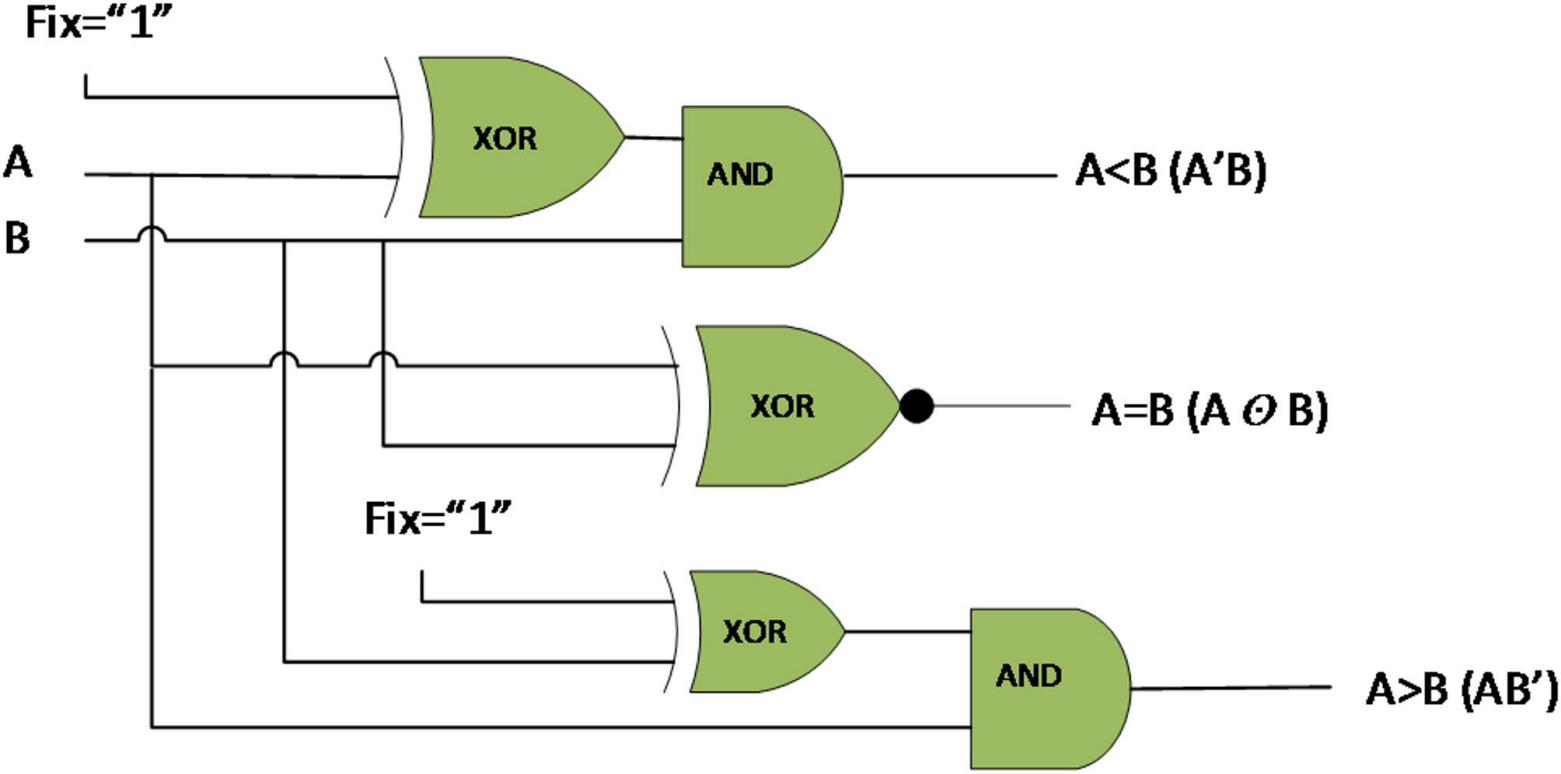
Logical diagram of single-bit comparator.

The diagram of a logical circuit for the single-bit comparator is shown in Fig. 5. It should be mentioned that the XOR gate with one input stuck at one is considered a NOT gate.

**Fig. 6:**
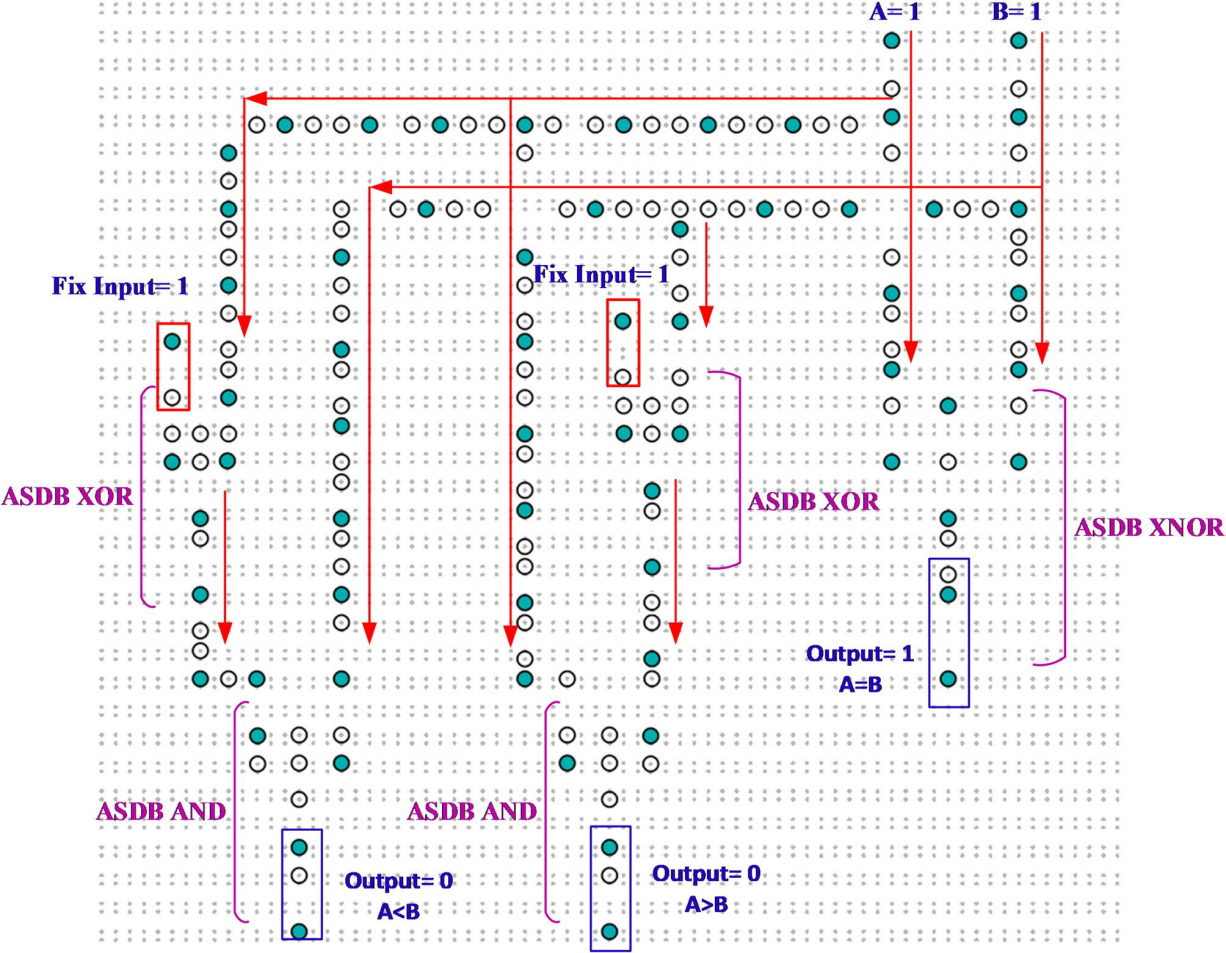
The ASDB layout of the single-bit comparator using the proposed gates.

The proposed ASDB layout for the single-bit comparator is given in Fig. 6. It’s worth mentioning that crossing the wires has been realized by using the approach presented in^13^.

As shown in Fig. 5, the proposed ASDB single-bit comparator circuit, based on a Hammer-shaped pattern, consists of only 168 DB atoms and occupies 552.108 nm.

## Simulation results

In the preceding section, we employed simulation tools to evaluate the output stability of the suggested ASDB designs^13^. The default settings of the simulator engine are as follows.

- DB atom size(nm)= 0.0441.
- Anneal cycles= 10,000.
- Relative permittivity= 5.60.
- Initial temperature (k)= 500.
- Minimum temperature(k) = 2.
- Physical validity checks cycles = 10.

**Fig. 7:**
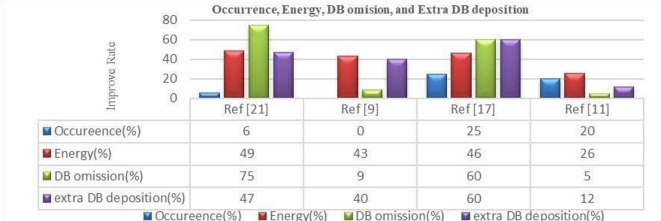
The improvement percentages of the proposed ASDB gates, in terms of occurrence, energy, DB omission, and extra DB deposition regarding^10,12,13,19^.

**Fig. 8:**
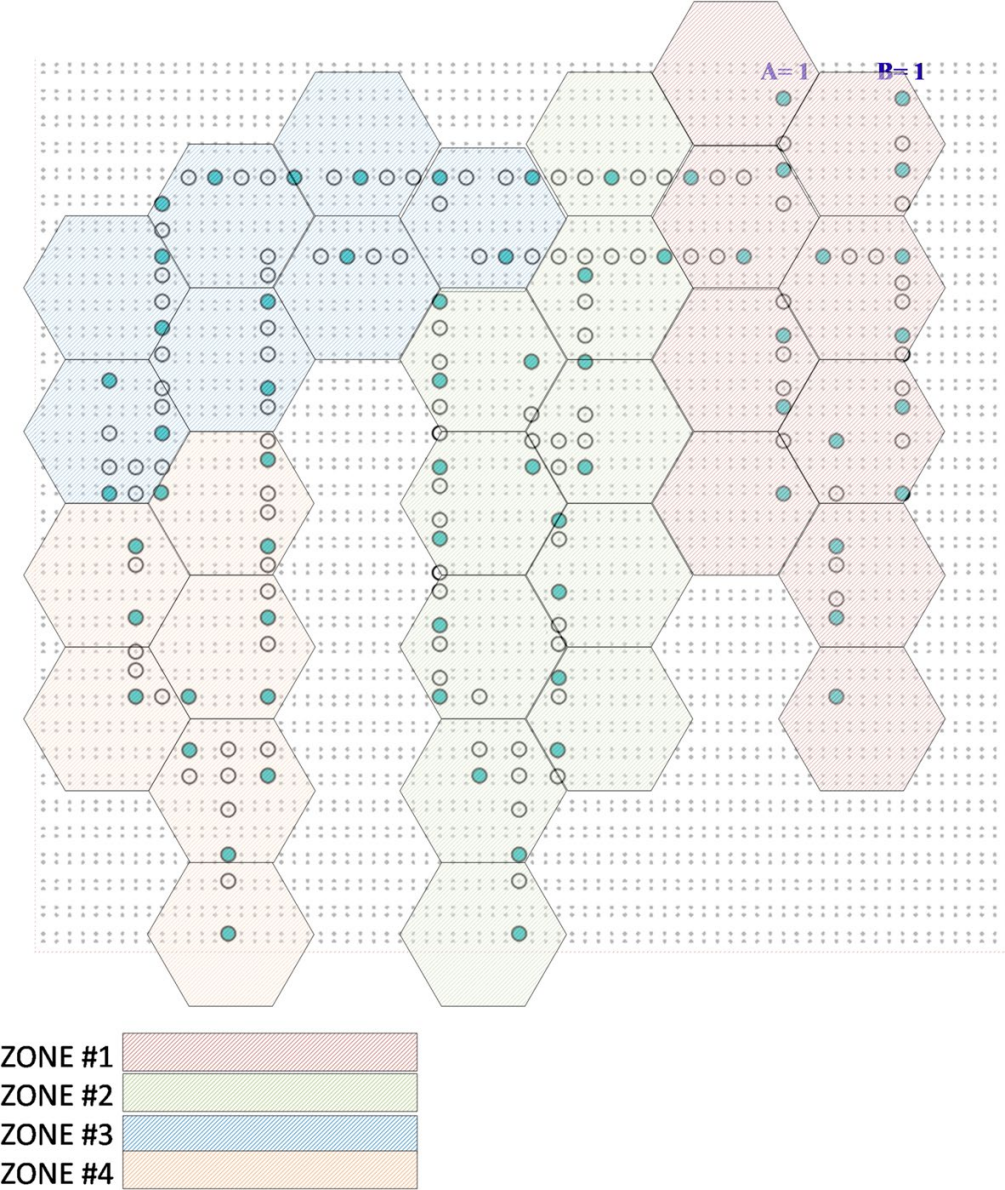
Dividing the proposed ASDB comparator into four parts for evaluating the robustness against possible defects.

The results obtained by simulating the proposed circuits can be reported as follows. Evaluating the occurrence and physical validity of the proposed ASDB gates, compared with previous works in^10,12,13,19^, are given in Table 2. Their energy analyses are also shown in Table 3. The obtained results show that the proposed Hammer-based ASDB gates show average improvements in occurrence in average 33%. Also, they have been better in energy consumption, by an average of –27%, compared with previous designs. Additionally, the suggested designs are physically valid for all input states. Figure 7 illustrates the average improvement percentage of the proposed design compared to previous works. The proposed basic gates have suitable conditions compared with previous works. Table 4 shows that the proposed single-bit comparator generates stable outputs for different input vectors while consuming less energy. Also, it can be observed that the proposed comparator has an acceptable occurrence regarding different inputs. Moreover, to investigate the robustness of the proposed structures, we have investigated them against defects such as DB omission and extra DB deposition. The results of evaluating the resistances of proposed ASDB gates are illustrated in Table 5. As can be seen, the proposed ASDB gates exhibit superior robustness against DB omission and extra DB deposition defects. They show improvements in average 56% in terms of DB omission and also show improvements in average 51% in terms of extra DB deposition, compared to designs reported in^10,12,13,19^. Also, the output stabilities of the proposed ASDB gates are examined against temperature variations and given in Table 5. The temperature variation is countered as one of the critical challenges in logical and computational circuits. The results indicate that the proposed basic gates have suitable temperature ranges for activity between 2 K and 500 K. Temperatures above 600 K may have a direct negative effect on circuit accuracy. Finally, to evaluate the proposed circuit against the possible defects, we divided the circuit into four parts, as shown in Fig. 8, and evaluated each one in terms of DB omission and extra DB deposition. According to Table 6, the results of the evaluation show that the proposed circuit is robust against possible defects in the different inputs. Furthermore, Table 7 highlights the robustness of the main circuit, with fault-tolerant performance ranging between 38% and 50%, indicating its stable behavior in the presence of possible defects.

**Table 2:** Comparing the result of occurrence evaluation and physical validity of the proposed ASDB gates; compared with previous works^10,12,13,19^.

ASDB gates	Proposed design		Design in^10^		Design in^19^		Design in^13 ^		Design in^12^	
	Occurrence (%)	Physical validity	Occurrence (%)	Physical validity	Occurrence (%)	Physical validity	Occurrence (%)	Physical validity	Occurrence (%)	Physical validity
AND	97	Yes	92	Yes	98	Yes	97	Yes	98.	Yes
NAND	89	Yes	89	Yes	77	Yes	89	Yes	79.	Yes
OR	99	Yes	97	Yes	61	Yes	98	Yes	65.01	Yes
NOR	82	Yes	93	Yes	NR	Yes	NR	Yes	NR	Yes
XOR	95	Yes	92	Yes	83	Yes	81	Yes	85	Yes
XNOR	97	Yes	NR	Yes	62	NR	79	Yes	68.	Yes

**Table 3:** Comparing results of the proposed ASDB gates in the field of energy with the previous designs^10,12,13,19^.

ASDB gates	Proposed design	Design in^10^	Design in^19^	Design in^13 ^	Design in^12^
	Energy (eV)	Energy (eV)	Energy (eV)	Energy (eV)	Energy (eV)
AND	0.128	0.501	0.528	0.599	0.401
NAND	0.281	0.509	0.475	0.580	0.410
OR	0.303	0.373	0.412	0.544	0.372
NOR	0.342	NR	NR	0.510	NR
XOR	0.328	0.510	0.510	0.512	0.393
XNOR	0.332	0.512	0.628	NR	0.422

**Table 4:** Evaluating results of four important parameters of the proposed ASDB comparator in all States of inputs.

Inputs		Evaluation parameters			
A	B	DB Atoms	Occupied Area ($\:\text{n}{\text{m}}^{2}$)	Occurrence (%)	Energy (eV)
0	0	157	552	77	1.034
0	1	157	552	73	0.984
1	0	157	552	68	0.891
1	1	157	552	79	1.038

**Table 5:** Comparing the robustness of the proposed ASDB gates against possible defects with previous works^10,12,13,19^.

ASDB gates	Proposed design		Design in^10^		Design in^19^		Design in^13^		Design in^12^	
	DB omission (%)	DB deposition (%)	DB omission (%)	DB deposition (%)	DB omission (%)	DB deposition (%)	DB omission (%)	DB deposition (%)	DB omission (%)	DB deposition (%)
AND	48	56	22	42	21	35	22	33	28	42
NAND	54	65	44	44	35	28	33	33	55	55
OR	44	41	22	42	22	28	22	22	28	42
NOR	67	60	33	55	NR	NR	NR	NR	66	66
XOR	68	63	66	66	55	55	66	42	77	77
XNOR	54	54	55	66	NR	NR	NR	NR	NR	NR
Average	56	55	40	53	22	24	36	33	51	56

NR = Not Reported.

**Table 6:** Evaluating the stability of the proposed gates with varying temperature (k) ranges.

ASDB Gates	Temperature (k) ranges A		Occurrence (%)	Temperature(k) ranges B		Occurrence (%)	Temperature(k) ranges C		Occurrence (%)	Temperature(k) ranges D		Occurrence (%)	Temperature(k) ranges E		Occurrence (%)
AND	2	500	97	500	600	48	600	700	34	700	800	13	800	1000	2
NAND	2	500	89	500	600	43	600	700	34	700	800	9	800	1000	0.36
OR	2	500	99	500	600	46	600	700	28	700	800	9	800	1000	1
NOR	2	500	82	500	600	32	600	700	23	700	800	9	800	1000	1
XOR	2	500	95	500	600	38	600	700	19	700	800	8	800	1000	4
XNOR	2	500	97	500	600	46	600	700	16	700	800	6	800	1000	7

**Table 7:** Evaluation results for the proposed comparator, in terms of DB omission and extra DB deposition defects.

A	B	ZONE#1		ZONE #2		ZONE #3		ZONE#4	
		DB omission	Extra DB deposition	DB omission	Extra DB deposition	DB omission	Extra DB deposition	DB omission	Extra DB deposition
0	0	45%	40%	42%	38%	48%	28%	40%	38%
0	1	40%	40%	45%	30%	55%	48%	40%	45%
1	0	48%	35%	52%	42%	35%	38%	48%	40%
1	1	45%	45%	50%	42%	38%	45%	40%	42%

## Conclusion

This paper introduced a new design pattern for atomic silicon dangling bond technology, the so-called Hammer-Shaped pattern. It has been used to design several basic gates like AND, OR, NAND, NOR, XOR, and XNOR, adding a one-bit comparator circuit for IoT applications. The proposed gates showed average improvements in average 33% for occurrence and 27% for energy efficiency, compared with similar designs for prior works shown in [9,11,17,21]. In addition, the proposed gates were tested against potential defects and temperature variations, which are crucial for the reliability of IoT devices operating under various environments. The proposed structures robustness results showed that the average improvements were 56 concerning DB omission defects and 51% concerning extra DB deposition defects, compared to related designs [9,11,17,21]. As for the one-bit comparator, it demonstrated an average robustness of 43% against the probable defects of DB omission and extra DB deposition. Moreover, regarding temperature, the proposed gates provided suitable outputs between 200 and 500 K, hence functioning within various IoT applications. As future work, we can apply the proposed Hammer-Shaped pattern in the design of more complex circuits, such as full adders, multipliers, and ALUs, for further capability in IoT systems and contribute toward the development of efficient, robust, and scalable printing devices within the IoT ecosystem.

## Data Availability

All data supporting the findings of this study are included in the article. Additional data are available upon reasonable request from the corresponding author, Mohammad Mosleh.
